# Urinary Exosomes

**DOI:** 10.1100/tsw.2009.128

**Published:** 2009-10-14

**Authors:** Irena Dimov, Ljubinka Jankovic Velickovic, Vladisav Stefanovic

**Affiliations:** Institute of Immunology, Institute of Pathology, and Institute of Nephrology, Faculty of Medicine, University of Nis, Serbia

**Keywords:** exosome, urine, proteomics, kidney disease, biomarkers

## Abstract

Exosomes are nanovesicles of endocytic origin that are secreted into the extracellular space or body fluids when a multivesicular body (MVB) fuses with the cell membrane. Interest in exosomes intensified after their description in antigen-presenting cells and the observation that they can significantly moderate immune responses *in vivo*. In the past few years, several groups have reported on the secretion of exosomes by almost all cell types in an organism. In addition to a common set of membrane and cytosolic molecules, exosomes harbor unique subsets of proteins, reflecting their cellular source. Major research efforts were put into their surprisingly various biological functions and in translating knowledge into clinical practice. Urine provides an exciting noninvasive alternative to blood or tissue samples as a potential source of disease biomarkers. Urinary exosomes (UE) became the subject of serious studies just a few years ago. A recent large-scale proteomics-based study of normal UE revealed a myriad of proteins, including disease-related gene products. Thus, UE have valuable potential as a source of biomarkers for early detection of various types of diseases, monitoring the disease evolution and/or response to therapy. As a relatively new field of research, it still faces many challenges, but UE have already shown some straightforward potential.

## INTRODUCTION

Exosomes are nanovesicles released by cells upon fusion of multivesicular bodies (MVB) with the plasma membrane. The first description of exosomes can be attributed to the biochemist Rose Johnstone, reporting in the 1980s on lipid-encased particles, 30–100 nm in diameter, produced as a mechanism for receptor down-regulation of specific membrane functions during reticulocyte maturation[1]. The interest in exosomes has recently spread, as it was proven that these vesicles are involved in a wide spectrum of physiological events, as alternative tools of immune system modulation, intercellular communication, and paracrine functions; or as pathogenic pathways in viral and prion-related diseases[2]. The variety of exosome functions could lead to their potential valuable role in the clinical application in many fields of medicine.

Urinary exosomes (UE) became the subject of extensive studies just a few years ago. Considering their origin, UE may provide a novel noninvasive method of acquiring unique information about the physiological or pathophysiological state of cells of origin. Furthermore, exosome isolation could result in remarkable enrichment of low-abundance proteins that have potential pathophysiological significance. Considering exosomes as unique carriers of mRNAs, microRNAs, and other active molecules involved in intercellular signalization[2], studies focused on UE could initiate novel approaches in clinical research. The ultimate goals are sensitive and specific biomarkers that can provide the clinician with simple, safe, and accurate tests in the follow-up of progression of disease and monitoring the response to therapy.

This review will focus on properties that define exosomes, summarizing the present knowledge of their biogenesis, functions, and their potential role in biomarker discovery. Some recent advances, but also still-open questions and challenges in this area, will be described.

## BIOGENESIS OF EXOSOMES

The endosomal system of the cell plays an important role in efficient segregation of endocytosed molecules destined for recycling from those that will be degraded by lysosomes[3,4]. It was shown that, in addition to classical exocytosis, an alternative endocytotic pathway has a role in cellular secretion. The main components of this alternative pathway are MVB. MVB were described in 1959 by Sotelo and Porter[5]. The most important step in the formation of MVB occurs in the endosome when its membrane invaginates into the lumen of this organelle to form intraluminal vesicles (ILV)[6]. MVB have a different fate: they can fuse with lysosomes, they can mature into the lysosomes, or can fuse with the cell membrane and release the ILV in the extracellular space, in which case the released ILV are called exosomes[7,8]. The regulatory factors that determine which one of these processes will occur are still not completely clear. Exosomes could be defined as secretory ILV of MVB, derived from the limiting membrane of the endosomes. During the process of membrane invagination, a certain quantity of cytoplasm is trapped inside the exosome. In the process of their formation, the exosomal membrane is reversed compared to the membrane of MVB and thus has the same topology as the cell membrane**.** This contrasts with endosomes, which are cytoplasmic-side out oriented[9].

The mechanisms involved in the formation of MVB and of protein sorting inside ILV are also not completely understood. The different studies point to the possible role of the ubiquitinization (mono- or oligoubiquitinization) and ESCRT (endosomal sorting complex required for transport) protein complex in this process[6,10]. ESCRT is a protein machinery consisting of ESCRT-0, -I, -II, and -III[11]. ESCRTs are predominantly cytosolic proteins that become recruited by endosomes. It was reported that ESCRT-0, -I, and -II have ubiquitin binding domains and play a central role in cargo sorting. It was shown that the depletion of the tumor susceptibility gene-101 (TSG101), a component of the ESCRT-I complex, has an inhibitory effect on receptor degradation and causes different endosomal morphology[12]. However, some data suggest that certain molecules, such as epidermal growth factor receptor (EGFR) and EGF, could be involved in the ESCRT-II–independent sorting pathway[11]. ESCRT-III has no ubiquitin binding domains, and it is probably important in the recruitment of deubiquitinizing enzymes that should remove ubiquitin before cargo incorporation into ILV. Another important role of ESCRT-III is to activate the molecular machinery that will facilitate the disassembly of the ESCRTs from the endosomal membrane[11,13]. The dissociation and recycling of the ESCRT complex is dependent on interaction with AAA-ATP-ase Vps4[8].

The mechanisms involved in the budding of vesicles from the limiting membrane of MVB are also poorly understood. It is known that ESCRT-III forms a lattice-like structure on the surface of the endosomal membrane[6]. Activation for assembly into lattices may be the consequence of interaction with the membrane itself, ALIX (accessory protein of the ESCRT-II), and some other ESCRT-III–related proteins. The presumption is that these lattices spatially restrict membrane curvature–inducing factors to initiate budding away from the cytoplasm. However, some authors point to the crucial role of the lipids in this process, such as phosphatidylinositol-3-phosphate ($PI3,5P_2$) and lysobisphosphatidic acid (LBPA)[14,15].

On the example of transferrin receptors in reticulocytes, it was shown that ubiquitinization is not a condition *sine qua non*[16], and some recent experiments on oligodendrocytes and melanocytes suggest that exosome biogenesis can be ubiquitin- and ESCRT-independent[17,18].

The tetraspan protein family is abundant in exosomes and may be responsible for recruitment of membrane proteins into ILV[8]. It is also possible that there could be differences in the mechanism of exosome secretion between the different cell types[9]. Hence, several different mechanisms may be involved in the process of ILV formation. This raises the question whether MVB from one cell releases different subpopulations of exosomes, or if there are distinct MVB subpopulations with rather uniform intrinsic ILV[8,9].

In recent years, scientists have focused their attention on the targeted profiling of the proteins present in exosomes. It was shown that exosomes mostly contain proteins that are present in the endosomal/lysosomal compartment of the cell[19]. The most common of these proteins are Hsp70 and Hsp90, tetraspan proteins (CD9, CD63, CD53, CD81, CD82), and MHC I molecules. Nearly all exosomes contain integrins, tubulin, actin, actin-binding proteins, and annexins (I, II, V, VI). Rab5, Rab7, ARF, and annexins have important roles in membrane transport/trafficking. Exosomes are also enriched in proteins that participate in vesicle formation, such as LBPA-binding protein and ALIX[2,10]. However, exosomes do not contain lysosomal proteases and other soluble endocytotic residents and any subunits of ATP-ase[4,19,20].

The protein structure of exosomes also depends on the cell type and it is, therefore, highly specific. The first described exosomes were from the maturing reticulocyte. During this process, TfR, glycophosphatidylinositol-anchored proteins, and integrin $\alpha 4\beta 1$ enter the endocytotic pathway, which results in the formation of exosomes and their release from the reticulocyte. In cytotoxic T lymphocytes, it was found that lysosomes often contain TCR, CD3, and CD8 in their granules. Antigen-presenting cells secrete exosomes that contain peptide-loaded MHC I and II molecules. Exosomes with specific protein markers were also found in culture supernatants of many other cells, such as mast cells, fibroblasts, Schwann cells, and others[4]. Exosomes are released from specific, selected sites on the cell surface. The "docking-fusion" events with the plasma membrane are probably mediated with Soluble N-ethylmaleimide–sensitive fusion proteins Attachment Protein Receptors (SNARE) and synaptogamin family members[8].

At first, it was thought that the role of exosomes was in the receptor down-regulation. Further studies in this field showed that the exosomes have a myriad of different functions in health (antigen presentation, immune response stimulation or suppression, activation of molecules and mRNA shuttling, etc.) or disease (the transmission and persistence of infections, promoting inflammation in autoimmune diseases, tumor surveillance)[2,4,19,20,21].

## PROTEOMICS APPROACH TO STUDIES OF EXOSOMES

Compared to the techniques required for other protein enrichment for proteome-based analysis, the isolation of exosomes may be fairly straightforward because it does not require sample concentration or cell lysis. Their protein and lipid contents could be identified and characterized unaffected by isolation and purification procedures[9].

Currently, the most effective method for exosome isolation is ultracentrifugation[9,22]. To further purify the exosomes, methods such as sedimentation via sucrose gradient ultracentrifugation and immunoisolation via antibody-based derivatized Dynabeads have proven effective[9,20,26]. The purified exosomes could be verified onwards usually using electron microscopy techniques or western blotting using antibodies against known exosomal biomarkers, such as hsp70, hsp90, and annexins I, II, V, VI[9,26]. The purified exosomes for proteomic analysis could be submitted to various protein or peptide separation approaches in combination with different types of mass spectrometry (MS) instruments[9].

Alternative approaches using ultrafiltration show some promise, but the main obstacle is a tendency to retain and concentrate soluble proteins in urine in addition to exosomes. These contaminating proteins are able to compete with exosomal proteins for identification by liquid chromatography (LC)-MS/MS and therefore may reduce the sensitivity of the discovery process[24].

## LIPIDOMIC APPROACH TO STUDIES OF EXOSOMES

Rapid technological advancements in MS and chromatographic techniques have led to expansion of lipidomics research. The increasing importance of this research field reflects the wealth of information accumulated in the past decade, which resulted in online resources such as Lipidomics Expertise Platform (http://www.lipidomics-expertise.de), Nature Lipidomics Gateway (http://www.lipidmaps.org), Lipid Bank (http://lipidbank.jp), Lipid Library (http://www.lipidlibrary.co.uk), Lipid Data Bank (http://www.caffreylabs.ul.ie), Kyoto Encyclopedia of Genes and Genomes (KEGG) (http://www.genome.jp/kegg/), European Federation for the Science and Technology of Lipids (http://www.eurofedlipid.org). Current research tends to shift to characterizing global changes in lipid metabolism in a system-integrated context[27,28].

Among other molecules, exosomes seem to be vehicles of bioactive lipids and lipolytic enzymes[2,29]. Advent soft ionization technologies, such as matrix-assisted laser desorption/ionization (MALDI), electrospray ionization (ESI), and atmospheric pressure chemical ionization (APCI) for MS, possibly coupled to LC, provided powerful tools for rapid and sensitive analysis of the majority or a substantial fraction of lipids possible in one analysis[27]. It seems that lipids involved in exosome composition differ according to the cells of origin and their function. Reticulocyte-derived exosomes showed no increase in cholesterol/phospholipids ratio, opposite to the MHC II–enriched exosomes derived from B cells[30,31]. Mast cell (RBL-2H3)– and dendritic cell–derived exosomes showed a high amount of disaturated phosphatidylcholine and phosphatidylethanolamine classes. This, together with a limited amount of diglycerides, suggests elevated membrane rigidity and an elevated transmembrane movement of lipids compared to the plasma membrane[30]. Most of the exosomes studied showed enrichment in raft lipids, such as cholesterol, sphingolipids, ceramide, and glycerophospholipids with long and saturated fatty-acyl chains[16,17,29,30].

Exosome formation could be a way to secrete enzymes involved in lipid signaling[29,30]. It was shown that cross-linking of sphingomyelin triggers calcium influx and ERK phosphorylation, indicating that these domains could be a specific signaling platform[30]. Recent studies provided evidence that the ESCRT-independent pathway of exosomal biogenesis requires ceramide and that release of exosomes was reduced after the inhibition of neutral sphingomyelinases[16,17].

The lipidomic approach would not only provide insight into the roles of specific lipids in exosome biogenesis and functions, but also holds promise in the biomarker discovery field, shoulder to shoulder with other powerful “omics” technologies.

## URINARY EXOSOMES

UE, as any other exosomes, are small intraluminal vesicles (<100 nm in diameter) that originate from MVB. They are delivered to the urine when the outer membranes of MVB fuse with the apical plasma membrane of the cell. Proteomic analysis of urinary vesicles through nanospray LC-tandem MS identified numerous protein components of MVB, suggesting their similar biogenesis as in other cell types[19,22,26]. Approximately 75% of the proteins that constitute ESCRT-0, -I, -II, -III, and ATP-ase complexes involved in the MVB formation are identified in UE, as well as ALIX[26].

UE are normally secreted into the urine from all cell types that face the urinary space and account for 3% of the total urinary protein[26,32]. Proteomic analysis identified specific membrane proteins starting from podocytes through transitional epithelial cells lining the urinary drainage system, including type A and B intercalated cells[24,26,32,33].

First, proteomic analysis of UE using tandem MS from normal human subjects revealed 295 unique proteins in UE. At least 20 of these proteins were already established as highly deregulated in various renal diseases and hypertension[22]. A recent study of UE from normal human urine using highly sensitive LC-MS/MS based on an ion trap mass spectrometer (LTQ; Thermo-Finigan; ThermoElectron, San Jose, CA) identified 1412 unique proteins. Of these proteins, 927 were not previously identified, including 14 phosphoproteins. The identified phosphoproteins are known to be involved in serine/threonine and tyrosine kinase signaling pathways, as well as in some other signaling pathways that occur during cytokine and growth factor receptor activation and numerous other cell regulatory processes**.** Further analysis of all obtained data identified 1132 proteins unambiguously. The full list of exosome proteins is publicly available at: http://dir.nhlbi.nih.gov/papers/lkem/exosome/[26].

A large number of identified proteins were soluble cytoplasmatic proteins and integral membrane proteins. Integral membrane proteins predominantly represent apical transporters present in every renal tubule segment. Multiple small GTP binding proteins, including proteins in the Rab, ARF, Rho, and Ral families, were identified, as well as cytoskeletal motor and peripheral-membrane proteins[26,33]. Ubiquitin was identified throughout the molecular weight range of the analysis, although the published data still do not distinguish between mono- and polyubiquitinated proteins[26].

## IMPORTANCE OF URINARY EXOSOMES IN CLINICAL RESEARCH

In the last years, the literature was overwhelmed with papers proving the role of exosomes (especially tumor and APC exosomes) in the transfer cognate receptors to homologous and heterologous cells. Their specific structure probably made them a more advantageous carrier of "distant" signal delivery compared to soluble molecules[2,34]. The lipid bilayer highly enriched with cholesterol, sphingomyelin, and other characteristic molecules may significantly contribute to the preservation of stabile conformation conditions, including post-translational modification of the proteins they carry, allowing their detection by powerful proteomic-based strategies. A number of the novel putative markers for the clinically important states were proven to be proteins that have undergone disease-specific post-translational modification[35]. One of the most important post-translational modifications is phosphorylation that regulates cellular signaling processes and may determine protein structure, function, and subcellular localization. Phosphoproteins and even the specific phosphorylation sites were identified in UE[26]. Thus, UE may become a very fruitful source for biomarker discovery. The valuable information that could be obtained from the proteomic, lipidomic, and other powerful molecular analyses also could provide further insight into the biogenesis and function of exosomes.

One of the possible applications in clinical research is large-scale biomarker discovery, such as those currently being pursued in blood, tissue homogenates, and liquor[24,33]. Another promising approach could be choosing a combination of proteins whose identification can be hypothesized to provide the required specificity (lack of false-positives) and sensitivity (lack of false-negatives). According to current guidelines, candidate biomarker discovery experiments would be done on samples from a relatively small number of extremely well-characterized patients compared to appropriately chosen control subjects[36]. Urine has evolved as one of the most attractive body fluids in the biomarker discovery field, with a potentially rapid application in the clinic. Considering their origin, UE may provide a novel noninvasive method of acquiring unique information about the physiological or pathophysiological state of the renal cells of their origin.

For the proteomics-based approach, exosome isolation could be helpful in minimizing highly abundant proteins in urine. This could also provide a significant enrichment of low-abundance urinary proteins that have potential pathophysiological significance and enhance the detectability of rare proteins that may have diagnostic value. Many UE proteins that may have diagnostic value were found to be markedly enriched (at least 30-fold) compared to kidney homogenate from the cortex, outer medulla, and inner medulla[37].

## Functions of Urinary Exosomes

A small glycosylphosphatidylinositol-anchored molecule, CD24, abundant in UE, was not detectable in previous proteome approach experiments[35,37,38]. This molecule, believed to be involved in cell-cell adhesion and signaling, suggests that the function of UE, as elsewhere in the body, may be beyond that of exocytic cell waste elimination. Furthermore, excretion via exosomes probably requires a significant amount of energy for formation, which might suggest that they were preserved through the evolution due to their other functions[1,39,40]. Exosomes are the newest family member of "bioactive vesicles" that function to promote intercellular communication. Exosome-mediated transfer of mRNAs and microRNAs could be one of the mechanisms of genetic exchange between cells[41]. One of the possible roles of the exosomes in the urinary tract is to regulate the cofunctioning between different parts of the nephron, through secretion and reuptake of their contents, most importantly mRNA and microRNA molecules, that can affect the function of the recipient cell[39].

Common excretion patterns of certain proteins or their fragments in various diseases could offer critical insight in the pathogenesis mechanisms[42]. Large-scale proteomics and phosphopeptidomics revealed, to date, 177 disease-related proteins, according to their presence in the Online Mendelian Inheritance in Man (OMIM) database. Of these candidate biomarkers, only 34 are associated with renal diseases. Gene products involved in hypertension and coronary disease, autoimmune disorders, and amyloidosis were detected[26].

## Urinary Exosome Proteins as Biomarkers in Renal Diseases

The results indicate that exosome isolation from urine samples may provide an efficient first step in biomarker discovery[37,40,43]. Aquaporin 2 (AQP 2), which was proven to be delivered into urine by exosomes, is one of the most studied proteins in urine and represents a reliable example of a well-established urinary biomarker for diagnosis of several water-balance disorders, such as diabetes insipidus[44,45,46,47].

All of the predominant apical ion and water transporters along the renal tubule were detectable in UE[26]. A candidate biomarker of renal tubule damage, Na+/H+ exchanger isoform 3 derived from UE of patients with acute kidney injury (AKI), could be beneficial in differential diagnosis between acute tubular necrosis and other causes of AKI[48]. Exosomal fetuin-A was proposed as a potential biomarker of AKI, based on data from a rat model, which were further supported by Western blots on patients[49].

Analysis of human UE by MS and immunoblotting could provide information with regard to kidney genetic diseases. Both polycystin-1 and -2 were easily detected in human UE[26]. Polycystin-1 and -2 are the protein products of two genes responsible for autosomal-dominant polycystic kidney disease (ADPKD) Types 1 and 2, one of the most common monogenic diseases and the most common genetic disease leading to renal failure. These proteins are of low abundance or undetectable in kidney tissue homogenate, but easily detectable in UE[22,26]. Early identification of patients with ADPKD would allow early intervention, possibly with one or more of the therapeutic approaches under trial, such as V2 receptor antagonists[50]. Distal renal tubular acidosis Type I (RTA Type I) occurs as a primary inherited disorder or may be a result of multisystem diseases, such as autoimmune disorders[51]. Protein products of the SLC4A1 gene responsible for the autosomal-dominant form of RTA Type I, ATP6V1B1 and ATP6V0A4 genes responsible for autosomal-recessive form with and without sensorineural deafness, were detected in UE[26]. Immunoblot analysis of UE was able to differentiate two different types of mutations for the thiazide-sensitive Na-Cl cotransporter of the distal convoluted tubule. This approach could have the potential to become a very simple and useful diagnostic tool to detect and subclassify Gitelman's syndrome[52,53].

It was recently discovered that exosomes, among other intracellular proteins, contain transcription factors (TF) that could be identified[54]. TF were shown to be detectable in UE, but not in whole urine. Urinary exosomal TF could represent a new class of biomarkers for acute and chronic renal diseases and may offer insight into cellular regulatory pathways. UE obtained from two AKI models (cisplatin or ischemia/reperfusion) and two podocyte injury models (puromycin-treated rats and podocin/Vpr transgenic mice), as well as human urine obtained from patients with AKI, focal segmental glomerulosclerosis (FSGS), and matched controls, were first-time analyzed for TF. The activating transcription factor 3 (ATF3) and Wilms Tumor 1 (WT-1) were selected as potential sensitive markers of kidney injury. ATF3 was continuously detected in UE, showing a specific expression pattern in both animal models. Exosomal urinary ATF3 was detected earlier than serum creatinine. ATF3 was not detectable in normal human urine samples or in the urine sample of patients with chronic kidney disease. Urinary WT-1 was detected in animal models before significant glomerular sclerosis. Urinary WT-1 was detected in FSGS patients, but not in control urine samples. Urinary ATF3 may be a novel renal tubular cell injury biomarker for detecting early AKI, whereas urinary WT-1 may point to early podocyte injury[32].

An alternative novel approach for biomarker discovery lies in a recent discovery that exosomes constitutively contain mRNA[41]. Exosomal urinary mRNA could be subjected to RT-PCR followed by sequencing of the DNA products that may allow identification of specific mutations, providing us with a noninvasive diagnostic technique for some genetic diseases, such as ADPKD[54].

Shedding of tumor vesicles has been demonstrated in many different malignant cell types[1,23,55]. Vesicles produced and secreted by tumor cells were shown to contribute to the ability of tumor surveillance by escape from immune response[56], and contribute to angiogenic[2,55] and dissemination[56] processes. Other studies suggest that exosomes can indirectly stimulate the immune system[57]. Currently, exosomes are of enormous interest to oncologists and immunologists who are now using them in clinical trials as tumor-antigen bearers to trigger tumor rejection by the body[2,3]. Exosomes may become a novel important source of malignant cell–associated antigens commonly not available in biological fluids. Analyses of UE in patients with urinary drainage tract malignancies may prove beneficial in providing novel and more sensitive biomarkers for an early disease diagnosis and therapy response monitoring, where the diagnosis by serial biopsy is rarely possible.

Recently, the role of small Ras-like GTPases, known as Ral GTPases, was investigated in 10 different bladder cancer cell lines[58]. Ral proteins represent a distinct subfamily of Ras proteins. Ral proteins were overexpressed in bladder cancer cells, suggesting that Ral proteins or their downstream pathway mediators constitute important factors for bladder cancer progression and metastasis, which may provide targets for future therapeutic approach[58]. One of the Ral-dependent transcriptional targets is CD24. Expression of CD24 in upper urinary tract urothelial carcinoma was shown to correlate with the tumor stage and grade, as well as features of aggressive tumor behavior[59].

The proteomic-based study of the urinary microparticles from individuals with bladder cancer[60] revealed eight overexpressed proteins and one down-regulated protein, compared to the samples from healthy individuals. Among the overexpressed proteins, five were associated with the EFGR signaling pathway. The alpha subunits of GsGTP binding protein, resistin and retinoic acid-induced protein 3, were also elevated. These candidate biomarkers need to be validated in further studies. Fibroblast grow factor 2 (FGF-2), a potent angiogenic factor involved in tumor invasion that is overexpressed in variety of tumors including bladder cancer, is probably released by exosomes[61]. There is one UE biomarker validation study in prostate cancer (PCa)[62]. Novel markers for PCa are needed as current established markers, such as prostate-specific antigen (PSA), lack diagnostic specificity and prognostic value. Cancer-derived exosomes obtained from an animal PCa model offered novel possibilities for the identification of candidate biomarkers for PCa[63]. Study of human UE as a possible treatment response markers for PCa patients showed no significant treatment-related decrease in exosomal PSA and prostate-specific membrane antigen (PSMA). Interestingly, both PSA and PSMA were not detected in UE from the healthy urine donor population, suggesting that few if any exosomes arise physiologically from the prostate[62].

## Urinary Exosomes and HIV

Renal disease is a relatively common complication of HIV infection. Clinical manifestations include HIV-associated nephropathy (HIVAN), IgA nephropathy, cryoglobulinemia, amyloidosis, and a lupus-like immune complex glomerulopathy. The role of HIV in HIVAN is thought to be direct infection of tubular cells and glomerular epithelium, possibly triggering apoptotic pathways. Inflammatory cytokines, such as TNFα, IL-1, and IL-6, may also play a role in the pathogenesis of renal processes; in a similar way, they bring about the immune system destruction.

Transfer of infectious agents via exosomes has been proposed for the spread of retroviruses, including HIV[64]. According to results obtained on a study of HIV-infected macrophages, HIV may assemble in compartments with the characteristics of late endosomal multivesicular compartments. To date, it remained uncertain whether the processes of HIV budding from the cell surface directly and that into the internal MVB are identical processes[65,66]. Viruses accumulate in "exosome-like bodies" rich with nonviral proteins, such as tetraspannins, enveloped with lipid bilayer as other MVB. Upon fusion of the MVB with the plasma membrane, "infectious exosomes" would be released into the circulation, free to infect other distant cells, avoiding the normal recognition tools of the immune system due to multiple self-antigens expressed on the exosome surface[67].

Clinical management of HIV revolves around measurements of the patient's CD4+ T-cell account and the amount of virus in the patient's plasma. These measurements require regular blood draws, which are tiresome for patients, but also jeopardizing for clinicians. UE of HIVAN patients could be more suitable for further analysis of HIV-positive patients due to a higher protein concentration and conformation maintenance.

According to the viral transmission by "infective exosomes"[2,64,67], it may be possible to detect p24 and other viral antigens in the UE of HIVAN patients. Proteomic analysis of the immune system products and viral antigens bundled in the UE of HIV patients might reflect the state of disease *per se* and provide monitoring of the response to the antiretroviral therapy.

## CHALLENGES AND BARRIERS

There are three major steps required for the development of urinary biomarker assays for routine clinical application: the discovery of biomarker candidates, the subsequent validation in larger clinical studies, and, finally, the clinical implementation[38]. The preconditions for any of these are rigorous standard protocols for collection, processing, and storage of urine samples that would allow correct, comparable, and reproducible UE analyses[26].

Procedures that would preserve exosomes unaffected before their isolation from urine should be standardized[26]. There is still a critical need for development of protocols for collection and storage of urine samples. The experimental results demonstrated the importance of the use of protease and bacterial inhibitors. Samples without protease inhibitors showed a major loss of exosomal proteins or no signal at all[32]. Alternatively, it could be possible that protease inhibitors may not be needed for spot urine collections that would be processed or frozen immediately[33]. Storage at –80°C and extensive vortexing after thawing was proven to maximize the recovery of UE[32]. Recommended protocols for isolation and storage are available at: http://intramural.niddk.nih.gov/research/uroprot/. However, more work is needed in this area.

Before the urinary exosomal isolation steps, procedures that would remove tubular casts, cells, and urinary high-abundant proteins should be applied[33]. A particularly challenging problem is Tamm-Horsfall protein (uromodulin) removal. This protein tends to form vast fibrillar aggregates in urine, particularly at low temperature. These aggregates can penetrate the exosomes and prevent their efficient isolation and purification by centrifugation methods, interfering with successful MS or immunoblotting[32]. Applying reducing agents, such as dithiothreitol, has shown some benefit. This procedure is based on denaturation of the zone pellucida domains in the Tamm-Horsfall protein disabling the aggregation process[22]. The ultracentrifugation approach that is commonly used in exosomal isolation requires extensive instrumentation and long processing, making this method inefficient for large-scale studies[26]. Filtration methods, as more beneficial for clinical studies, have been proposed[32]. Possible alternative methods for isolation of UE, including adsorption, evaporation, dialysis, and/or immunoisolation[32], have to be evaluated. Currently, there is still a lack of standard protocols and there are few research groups trying to define optimal conditions for urine sampling, storage, and shipping, as well as exosomal isolation and purification as a prelude to biomarker discovery studies[24,26,32,62].

For the biomarker discovery, the UE proteomics approach is presently most oriented to detecting and identifying one or more proteins that are specifically up- or down-regulated in the affected population. This approach requires an extremely well-defined patient population, rigorous protocols, and an appropriate control group[33]. The major challenge could be development of quantification approaches that allow detection of changes in excretion rates of particular disease biomarker candidates. Creatinine as a normalization variable may not be adequate due to high individual variability in its rate of excretion. Alternatively, the excretion of specific exosomal proteins can be normalized by the excretion of common exosomal markers independent of their source, such as HSP 70, ALIX, or TSG101[22,32,48]. More studies are needed to evaluate these alternatives.

## CURRENT PERSPECTIVES

Like any new tool, exosome analysis has advantages and disadvantages that need to be explored prior to any exosome analysis project. Nevertheless, this approach holds promise as a noninvasive source of multiple disease biomarkers that could provide clinically useful information. There are some straightforward potential applications of exosome analysis that may prove to be of considerable value, particularly in the area of genetic mutation analysis[33,43]. Even without quantification, certain biomarkers may be useful if completely absent in the control subjects, but strongly positive in the patients or *vice versa*. This may be valuable in genetic diseases diagnostics, such as Bartter syndrome where the mutant protein form was shown to be entirely absent from UE[26].

Tandem MS may be applied for determination of the primary sequence of exosomal proteins for the identification of mutations and polymorphisms. New tandem mass spectrometers have superior abilities to carry out *de novo* sequencing of proteins[33]. A computational strategy that involves digestion of the target proteins with a battery of proteases, followed by analysis of the resulting spectra to assemble protein sequences for particular protein targets, was proposed[68].

A number of the novel putative markers for the clinically important states represent fragments of proteins or proteins that have undergone disease-specific post-translational modification[35]. Exosomes may significantly contribute to the maintenance of these subtle modifications, allowing their detection by powerful proteomic-based strategies.

## CONCLUSION

Like any new tool, exosome analysis has advantages and disadvantages that need to be overcome prior to any exosome analysis project. Given the noninvasive nature of the sample collection procedure and the large amount of information potentially available from such studies, it seems likely that exosome analysis will play a role in nephrology research in the future, shoulder to shoulder with the standard proteomic-based approach.

## ACKNOWLEDGMENTS

This work was supported by a grant, No. 145004, from the Ministry of Science and Technical Development of the Republic of Serbia.
